# Experimental protection of quantum coherence by using a phase-tunable image drive

**DOI:** 10.1038/s41598-020-77047-5

**Published:** 2020-12-10

**Authors:** S. Bertaina, H. Vezin, H. De Raedt, I. Chiorescu

**Affiliations:** 1 CNRS, IM2NP (UMR 7334), Institut Matériaux Microélectronique et Nanosciences de Provence, Aix-Marseille Université, 13397, Marseille, France; 2 CNRS, LASIRE (UMR 8516), Laboratoire de Spectroscopie pour les Interactions, la Réactivité et l’Environnement, Université de Lille, 59000, Lille, France; 3 Zernike Institute for Advanced Materials, University of Groningen, Nijenborgh 4, 9747 AG Groningen, The Netherlands; 4 Department of Physics, The National High Magnetic Field Laboratory, Florida State University, Tallahassee, FL 32310 USA

**Keywords:** Qubits, Condensed-matter physics, Quantum physics

## Abstract

The protection of quantum coherence is essential for building a practical quantum computer able to manipulate, store and read quantum information with a high degree of fidelity. Recently, it has been proposed to increase the operation time of a qubit by means of strong pulses to achieve a dynamical decoupling of the qubit from its environment. We propose and demonstrate a simple and highly efficient alternative route based on Floquet modes, which increases the Rabi decay time ($T_R$) in a number of materials with different spin Hamiltonians and environments. We demonstrate the regime $T_R \approx T_1$ with $T_1$ the relaxation time, thus providing a route for spin qubits and spin ensembles to be used in quantum information processing and storage.

## Introduction

In open quantum systems, coherence of spin qubits is limited by spin-spin interactions, spin diffusion, inhomogeneity of the static and microwave fields^1^ as well as charge noise^2^. An increase in coherence time is achieved by dynamically decoupling (DD) qubits from their surroundings using distinct Electron Spin Resonance (ESR) pulses^3–7^. However, such pulses have inherent imperfections and fluctuations, thus requiring their own layer of DD, resulting in a doubly dressed qubit. The technique of concatenated DD^8,9^ has been proposed for nitrogen vacancy (NV) centers up to the second order of dressing^8,10–12^. Here we demonstrate a pulse protocol based on Floquet modes which successfully increases the decoherence time, independently of qubit initial state, in a number of materials with different spin Hamiltonians and environments, such as low and high spin-orbit coupling for instance. Rather than focusing on decoupling from the bath by strong excitation, we use very weak pulses and alter the dynamics of the entire system. For short spin relaxation times accessible to our measurement setup (at around 40 K) one can do a direct comparison with the coherence time, and we demonstrate the regime $T_R\approx T_1$. In magnetic diluted systems $T_1\gg T_2$, e.g. $T_1$ of several *ms* in rare earth ions such as Y$_2$SiO$_5$:Er$^{3+}$^13^ and Y$_2$SiO$_5$:Yb$^{3+}$^14^ or $^{28}$Si:Bi with a tunable $T_1$ of thousand of seconds^15^. Our general method can thus lead to very long persistent Rabi oscillations, using a single circularly polarized image pulse.

The use of strong continuous microwave excitation has been proposed as a way to protect qubits^16,17^ although the quantum gates would need proper redesigning. In related studies, complex pulse design using an arbitrary waveform generator, proved essential in studying Floquet Raman transitions^18,19^ and quantum metric of a two-level system^20^ in nitrogen vacancy (NV) centers. It is worth noting that in the case of concatenated DD, the frequency of the second order ($n=2$) excitation has to match the Rabi frequency of the first excitation ($n=1$); also, the two excitations are linearly polarized and perpendicular to each other (the method extends to higher orders in *n*). Experimentally, the protocol quickly becomes complex and demanding in terms of pulse design and frequency stability, above the second order.

Our protocol uses two coherent microwave pulses: a main pulse drives the qubit Rabi precession while a low-power, circularly polarized (image) pulse continuously sustains the spin motion. The image drive has a frequency close to the main drive and its amplitude is 1-2 orders of magnitude smaller. In this way, a quantum gate could be driven by regular pulses, without the image pulse, while the time interval between gates could be filled with an integer number of Rabi nutations that use our protection protocol. Such scheme would protect the coherence of the qubit in-between usual quantum gates. We note that the initial phase difference between the two pulses allows to tune the spin dynamics by enhancing (or diminishing) the Floquet modes^21^ of its second dressing.

The protocol described here can impact quantum sensing of magnetic fields seen as a perturbation of a given Rabi oscillation. Its Fast Fourier Transform (FFT) has a width $\sim T_R^{-1}$ and the condition $T_R\approx T_1$ significantly improves the field resolution.

The technical implementation is simple and can be generalized to any type of qubit, such as superconducting circuits or spin systems. In this paper we focus on the experimental implementation and simply observe that numerical simulations based on Bloch model with $T_2=2T_1$ describe the final results very well.

## Results and discussion

**Figure 1:**
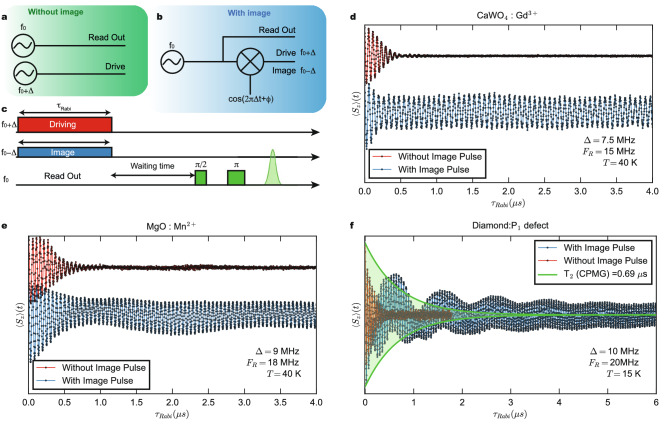
Comparison of Rabi oscillation with or without the image pulse. (**a**–**c**), Schematic of the microwave implementation. (**a**) The drive ($f+\Delta$) and the readout ($f_0$) sources are independent. (**b**) Using the same source as the readout ($f_0$), the drive pulse ($f_0-\Delta$) is generated by a non-linear mixing with a low frequency signal $\Delta$, with $f_0/\Delta \sim 10^3$, a process that creates a low-amplitude image drive ($f_0-\Delta$) as well. The drive, image and readout pulses are coherent and the phase relationship is tunable. (**c**) Pulse sequence: the drive and the image pulses act at the same time on the qubit while the readout is sensitive to the $S_z$ projection by a spin-echo process. (**d**–**f**) Rabi oscillation of the CaWO$_4$:Gd$^{3+}$, MgO:Mn$^{2+}$and P1 defects respectively in the presence (blue) or absence (red) of the image pulse at the optimum condition $F_R=2\Delta$. The green guideline in (**f**) shows the improvement of the coherence time when compared to a CPMG pulse sequence while the blue curve shows not only a long coherence but also beatings which are tunable via phase-tunable Floquet dynamics (see text).

The standard method to induce Rabi oscillations in a two-level system (TLS) is to apply an electro-magnetic pulse of frequency $f_0$ equal to the TLS level separation (resonance regime, $g\mu _B H_0=hf_0$, with $\mu _B$ the Bohr magneton, $H_0$ the static magnetic field and *h* the Planck constant ). The pulse will drive the spin population coherently between the two states. Experimentally, the drive is at a frequency $f_0+\Delta$ (where $f_0$ is the Larmor frequency and $\Delta$ is a small detuning away from the resonance condition) followed by read out pulses of frequency $f_0$ to record the state $\left\langle S_z\right\rangle$ (see Fig. 1a). The method introduced here makes use of two coherent microwave pulses (see Fig. 1b,c): the drive pulse at $f_0+\Delta$ of amplitude $h_d$ and length $\tau _{Rabi}$ creates quantum Rabi oscillations while the second one sustain them using a very low power image of the drive ($h_i\ll h_d$), operated at $f_0-\Delta$. In order to probe $\left\langle S_z(\tau _{Rabi})\right\rangle$ at the end of the Rabi sequence, we wait a time longer than $T_2$, such that $\left\langle S_x\right\rangle \approx \left\langle S_y\right\rangle \approx 0$, followed by $\pi /2- \pi$ pulses to create a Hahn echo of intensity proportional to $\left\langle S_z\right\rangle$ (Mn$^{2+}$ spins are readout without echo, as detailed in Sect. IIB of Supplementary Information). Figure 1b shows one way of coherently creating the drive and its image, by means of a mixer multiplying a pulse at frequency $f_0$ with an intermediate frequency (IF) cosine signal allowing to control the detuning $\Delta$, phase $\phi$ and the pulse length, shape and amplitude.

Rabi oscillations of three different types of paramagnetic systems—a rare earth ion (Gd$^{3+}$), a transition metal ion (Mn$^{2+}$) and a defect in diamond (P1)—are shown in Fig. 1d–f, respectively. Their detuned Rabi oscillations induced by the drive pulse only (red curves) are of similar frequency ($\approx 20$ MHz) and last for a small number of nutations ($<20$). The blue curve shows the Rabi oscillation when the image pulse is superimposed. The oscillations remain intense far beyond the decay time of the red curves and their number is dramatically increased. This effect has maximum impact when the frequency difference between the drive and the image pulses $2\Delta$ matches the Rabi frequency induced by the main drive, $F_R$. In addition of the very long coherence time, we observe a slow amplitude modulation, depending on the phase $\phi$ and attributed to Floquet modes, as explained below.

**Figure 2:**
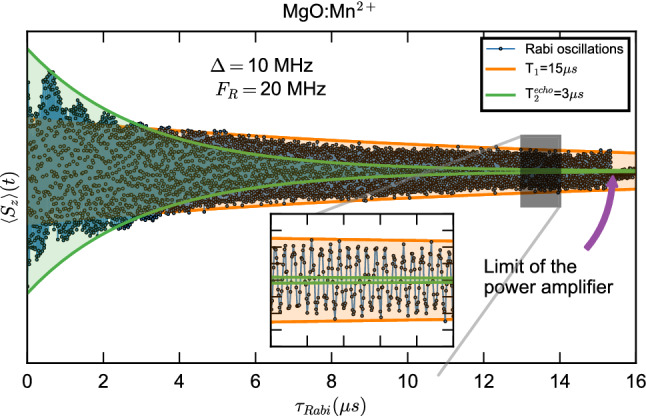
Rabi decay under image pumping condition at 40 K. The decay of the Rabi oscillations (green) for a Mn$^{2+}$ spin is limited by the relaxation time $T_1$ (orange) when an image pulse is used to sustain the dynamics. The inset shows the long-lived oscillations, all the way up to the 15 $\upmu$s limit as imposed by the power amplifier of the setup.

We study the new decay time of the Rabi oscillation under image pumping, by tuning the relaxation time via temperature control and by applying the longest drive pulse available to us (Fig. 2). The length of the drive pulse is limited by the pulse power amplifier of the setup, with a maximum pulse length of 15 $\upmu$s. At 40 K, the relaxation time of the spin system MgO:Mn$^{2+}$ ($S=5/2$) is also $\approx 15\,\upmu$s. The Rabi oscillation for the transition $+\frac{1}{2}\leftrightarrow -\frac{1}{2}$ is shown in Fig. 2. Guidelines showing the exponential decays due to $T_2$ (in green) and $T_1$ (in orange) measured by Hahn echo and inversion recovery respectively, are added as well. While the amplitude of the Floquet mode (slow amplitude modulation) decreases with $\sim$ $T_2$, the Rabi oscillations persists with a decay time $\approx T_1$.

### Protection of quantum coherence for different initial states

We analyzed our protocol for different initial states. For initial and final states along $+X$, $+Y$ and $+Z$ we observe that the protection protocol is effective for times up to the maximum amplifier gate length of 15 $\upmu$s.

#### Pulse sequence

**Figure 3:**
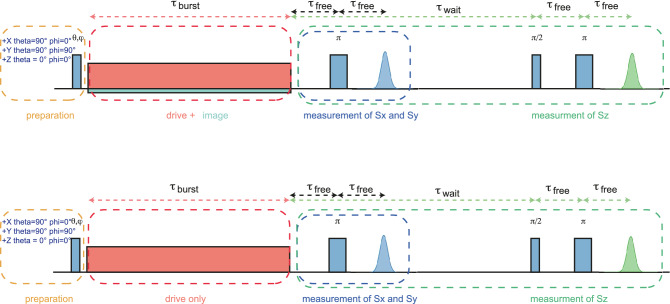
Pulse sequence used for different initial states. First, the spin is prepared in the ground state $+Z$ or, after a $(\frac{\pi }{2})_{y,x}$, in the $+X$ or $+Y$ states. A Rabi oscillation is performed during $\tau _{burst}$ for an integer number of Rabi flops, with or without the image pulse $h_i$ (top and bottom panels respectively). The final state is along the same direction as the initial one, and the readout is done using an usual echo detection (blue and green areas).

The protocol proposed here was tested for different initial states and spin systems. In the following, the details of the experiment are given. The ground state of the spin is along $+Z$ axis and it is used as initial state. In Fig. 3, this is shown in orange and labeled “preparation”. The $+Z$ state is obtained by thermalization. States $+X$ and $+Y$ are obtained from $+Z$ using a hard $\frac{\pi }{2}$ pulse around the *y* or *x* axes, respectively, able to excite the whole spin ensemble (or spectroscopic line). In MgO:Mn$^{2+}$and P1, this is ensured by their narrow spectroscopic linewidths $\Gamma$, while in CaWO$_4$:Gd$^{3+}$, $\Gamma$ is of the same order of magnitude with the maximum excitation bandwidth. Therefore, some Gd$^{3+}$ spins of the spin packet might not be in a perfect $+X$ or $+Y$ state. However, we didn’t notice any particular effect in the final results for Gd. We note that by combining rotations around any of the *x*, *y* and *z* axes, we can prepare the initial state in any position. Once prepared, the spin state is subjected to coherence protected Rabi protocol or to the usual Rabi drive for an integer number of Rabi flops (top and bottom “burst” panels in Fig. 3, respectively). Thus, the final state is along the same direction as the initial one^22^. The echo-based measurement for an initial state $+Z$ is shown in the green panel (with $\tau _{wait}\gg T_2$). For initial states $+X$ and $+Y$ (blue panel), one have to wait a time $\tau _{free}$ in order to let the spin packet defocus before applying a $\pi$ pulse and detect the subsequent echo signal.

Throughout this article, the $\pi /2$ and $\pi$ pulse lengths are 14 and 28 ns, respectively. The readout waiting times $\tau _{wait}$ are approximately 5 $\upmu$s for P1 defects, 6 $\upmu$s for Mn$^{2+}$ and 10 $\upmu$s for Gd$^{3+}$.

#### P1 defects in diamond

**Figure 4:**
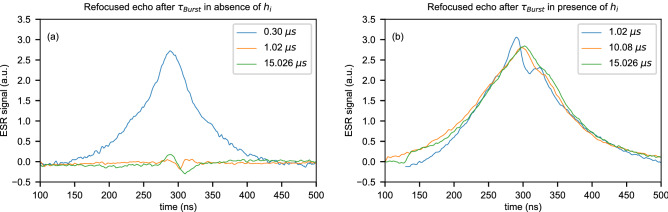
P1 defects in diamond. Echo signal for initial and final state along $+Z$ measured after a Rabi burst of various length. Without image pulse (**a**) the coherence is quickly lost after 0.30 $\upmu$s as shown by an absence of signal above 1 $\upmu$s. With protection protocol in place (**b**), the signal is well conserved up to maximum burst length of 15 $\upmu$s.

In the case of P1 defects in diamond, we present measurements of the spin echo signal when the initial and final states are along $+Z$, with and without the protection protocol for different lengths of the Rabi pulse $\tau _{burst}$. Without image pulse (Fig. 4a) the signal is visible after a Rabi burst of 300 ns, but it is rapidly lost for times longer than 1 $\upmu$s. With the protection protocol in place (Fig. 4b), the signal is almost entirely conserved for times up to 15 $\upmu$s. This behavior shows a significant improvement over the CPMG method (see Supplementary Information 1, $T_2=0.69$ $\upmu$s). For this experiment the temperature was set to $T=$15 K and $\tau _{free}=300$ ns.

#### CaWO

**Figure 5:**
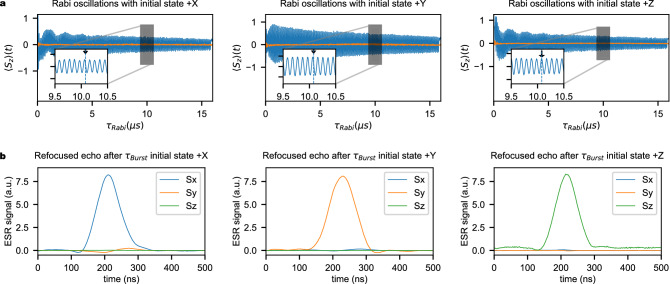
CaWO$_4$:Gd$^{3+}$. (**a**) Rabi oscillations shown as the real and imaginary part of the recorded signal (blue and orange lines, respectively). From left to right, the initial (and final) state of the spin are $+X$, $+Y$ and $+Z$. After a time given by an integer number of Rabi flops, shown with an arrow in the inset, (**b**) the spin-echo signal is recorded for each spin component, using the measurement sequence described in Fig. 3.

$_4$*:Gd*$^{3+}$ For Gd$^{3+}$ spins, we measured the spin-echo signal after an integer number of Rabi flops for initial/final states along the $+X$, $+Y$ and $+Z$ axes. The Rabi oscillations are shown in Fig. 5a as the real and imaginary part of the recorded signal (blue and orange lines, respectively). One notes that for the initial state along $+X$ and $+Y$ the Rabi signal starts and ends at zero value, with the end time being indicated by the arrow in each inset. Figure 5b shows large echo signals after 10 $\upmu$s, much longer than $T_{2CPMG}=4$ $\upmu$s (see Supplementary Information 1), for each of the initial state preparation. Using a combination of rotations around the *x*,*y* and *z* axes, we can create any initial state for the spin and thus use the image pulse protection for any arbitrary state. The experimental conditions for these measurements are: temperature $T=40$ K and $\tau _{free}=200$ ns.

### Qubit dynamics

The qubit dynamics in the absence of a bath, is described by the spin Hamiltonian in the laboratory frame (see Supplementary Information 1):

1 $$\begin{aligned} {\mathcal {H}}= f_0S_z+2h_{d}S_x\sin (\omega _+t+\phi )+2h_{i}S_x\sin (\omega _-t-\phi -\theta ) \end{aligned}$$

where $f_0$ is the Larmor frequency caused by the static field, $h_d$ and $h_i$ are the microwave drive and image field, respectively, $\frac{\omega _{+,-}}{2\pi }=f_0\pm \Delta$, $\phi$ is a tunable phase (see Fig. 1) and $\theta$ is a small additional phase, possibly created by imperfections of the setup (as discussed in Supplementary Information 1). Variables $f_0,h_{d,i}$ and $\Delta$ are expressed in units of MHz with $h_{d,i}\ll f_0,\omega _\pm$. After using the rotating wave approximation (RWA) for a rotation with $\omega _+$, the Hamiltonian (1) becomes:

2 $$\begin{aligned} \mathcal H_{RF}&=-\Delta S_z+h_{d}(S_x\sin \phi -S_y\cos \phi )\nonumber \\&\quad -h_{i}[S_x\sin (4\pi \Delta t+\phi +\theta )+S_y\cos (4\pi \Delta t+\phi +\theta )]. \end{aligned}$$

**Figure 6:**
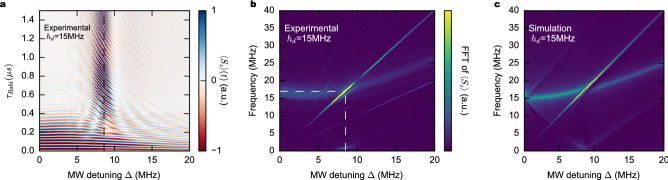
Rabi dynamics as a function of detuning at fixed drive level $h_d$. (**a**) Contour plot showing $S_z$ as a function of pulse length $\tau _{Rabi}$ and microwave detuning $\Delta$. At $F_R=2\Delta$ (dashed), the Rabi oscillations are optimally sustained by the image pulse. (**b**) The FFT of (**a**) shows an intense peak where the optimum protection is achieved (dashed lines). (**c**) A simulated FFT using the rotating wave approximation and the parameters of (**b**), is in agreement with the experimental observations.

When the image field $h_i$ is absent, the Eq. (2) has no explicit time dependence and the Rabi frequency is simply $F_R=\sqrt{\Delta ^2+h_d^2}$. When $h_i$ is present, the dynamics of $\left\langle S_z\right\rangle$ can be solved numerically, as it is shown in Fig. 6 for the case of CaWO$_4$:Gd$^{3+}$.

For a fixed power $h_d$ of the drive pulse, Rabi oscillations are measured as a function of the detuning $\Delta$. As shown in the contour plot of Fig. 6a, $\left\langle S_z\right\rangle (t)$ vanishes after few oscillations except when the condition $F_R\sim 2\Delta$ is met. At this Floquet resonance, $\left\langle S_z\right\rangle$ keeps oscillating for a very long time ($>15\,\upmu$s). Its Fast Fourier Transform (FFT) is presented in Fig. 6b. The free (unprotected) Rabi oscillations mode $F_R$ is rather weak and broad showing the large damping caused by the environment. However, when the mode crosses the frequency of the image pulse, indicated by the vertical white dashed line, the peak becomes intense and narrower, as the qubit protection from environment is activated. The condition $F_R=\sqrt{\Delta ^2+h_d^2}=2\Delta$ (or $h_d=\sqrt{3}\Delta$) gives the most efficient protection of the Rabi oscillation (see Supplementary Information 1).

The general condition is $F_R=n\Delta ,n=2k, k\in N$ showing a comensurate motion of the qubit and $h_i$ on the Bloch sphere. In other words, the qubit and the image field share a synchronous dynamics generated by the torques of the two drives (see also Sec.III D of the Supplementary Information 1).

We can compare the experimental result to the model described by the Hamiltonian (2) which can be rewritten as in Eq. (S14) in Supplementary Information : ${\mathcal {H}}_{RF}=-\Delta S_z+S_+[h_{d}e^{-i(\phi -\pi /2)}+h_{i}e^{i(4\pi \Delta t+\phi +\theta +\pi /2)}]$. When the “image” pulse is not applied, the Hamiltonian is time independent and the propagator is simply the matrix exponential of the Hamiltonian: $U_p(t)=\exp (-i2\pi {\mathcal {H}}_{RF} t)$. When the image pulse is present ($h_i>0$), the Hamiltonian becomes explicitly time-dependent. Although a second canonical transformation RWA could remove the time dependence if $\Delta \gg h_i$, it is importat to leave $\Delta$ as a free parameter since the methods works at resonance as well ($\Delta =0$). Thus, for the sake of generality, we solved numerically the explicit time-dependent differential equations using QuTIP^23^. The parameters used in the simulation have been measured independently: the microwave drive field $h_d$ has been calibrated using the frequency of Rabi oscillations at no detuning ($\Delta =0$), the image drive $h_i$ was measured by a spectrum analyzer directly connected to the output of the AWG ($h_i/h_d\approx 0.12$), relaxation ($T_1$) and decoherence ($T_2$) times were measured by inversion recovery and Carr-Purcell-Meiboom-Gill (CPMG) protocol, respectively (see Supplementary Information 1). We used QuTiP implementation of Lindblad’s master equation with $S_-$ as collapse operator which is equivalent to the phenomenological Bloch model for the case $T_2=2T_1$. Figure 6c shows the FFT of $\left\langle S_z\right\rangle (t)$ computed using the time evolution of ${\mathcal {H}}_{RF}$. The Hamiltonian (2) describes very well the protection of the coherence by means of the image pulse. Note the existence of a Floquet mode at $\Delta$=7.5 MHz of frequency $\sim 1$ MHz, visible in both the experimental and theoretical contour plots of Fig. 6.

**Figure 7:**
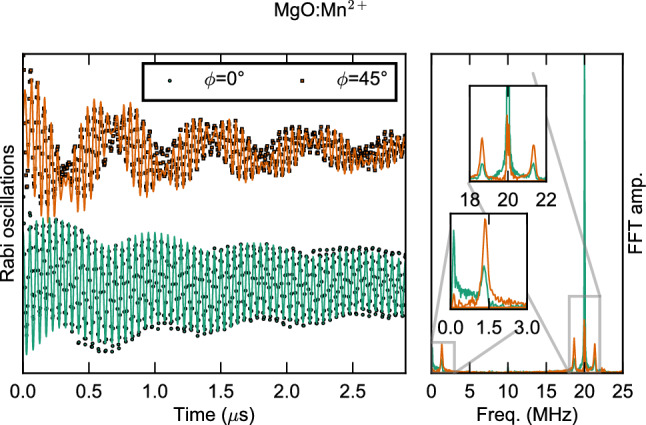
Phase-tunable Floquet dynamics. Rabi oscillations of Mn$^{2+}$ at 40 K for two values of $\phi$. For $\phi =0^\circ$ (green symbols), $h_d$ and $h_i$ pulses have the same initial phases resulting in optimally sustained Rabi oscillation. For $\phi =45^\circ$ (orange symbols), strong beatings are observed ($h_{d,i}$ terms of ${\mathcal {H}}_{RF}$ are $\perp$). The solid lines are simulations using our model with no fitting parameters. The right panel shows the experimental FFT traces. The green one has a well-defined peak at $F_R$ while in the other one two peaks (top inset) are separated by twice the Floquet mode frequency (bottom inset).

The Floquet mode appears as beatings of the Rabi frequency and is $\phi$-tunable. Similarly to the case of Gd$^{3+}$, the qubit protection and Floquet mode dynamics is obtained for the $S=5/2$ spin of MgO:Mn$^{2+}$, here measured under the experimental conditions of Fig. 2. Rabi oscillations and corresponding FFT spectra are shown in Fig. 7 for two values of the initial phase, $\phi =0^\circ$ (green) and $\phi =45^\circ$ (gold), while simulations are shown in black. The decay times are much larger than $T_2$ for both values (here $T_1\approx 15$ $\upmu$s, see Fig. 2); however, the dynamics is strikingly different. When the drive and image pulses have the same initial phase (one can consider the initial time in Eq. (2) as $-\frac{\theta }{4\pi \Delta }$ without loss of generality), the Rabi oscillations have maximum visibility, with almost no beatings (see Supplementary Information 1). At $\phi =45^\circ$, the spin torques generated by the $h_d$ and $h_i$ fields induce strong beatings or a Floquet mode creating two additional modes of the Rabi frequency. The left panel shows Rabi splittings equal to the Floquet frequency for $\phi =45^\circ$ and a single Rabi oscillation for $\phi =0^\circ$.

**Figure 8:**
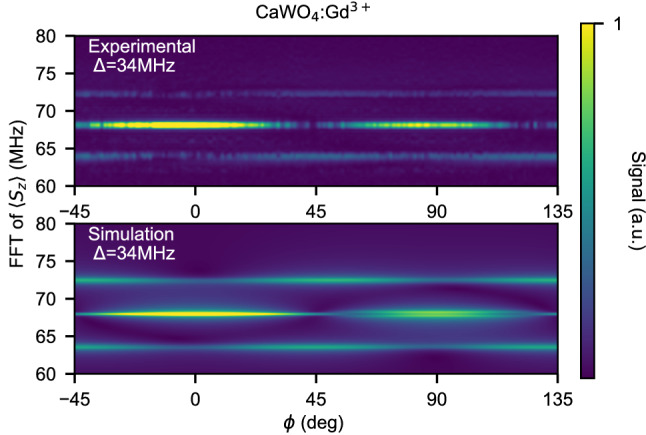
Phase-dependence of the Floquet mode. Experimental (top) and simulated (bottom) FFT of Rabi oscillations for $\Delta =h_d/\sqrt{3}=34$ MHz as a function of $\phi$ (amplitude is normalized to the highest peak). For $\phi =2k\pi /4, k\in N$, Rabi oscillations have a single mode $F_R$ while for $\phi =(2k+1)\pi /4$ a splitting of twice the Floquet mode frequency is observed. While its frequency is fixed, the intensity of the Floquet mode changes gradually, with a period of $\pi /2$.

Experimentally, we can continuously vary the value of $\phi$ and analyze the frequency and intensity of the Floquet mode. As an example, a comparison between theory and experiment is shown in Fig. 8 for the case of CaWO$_4$:Gd$^{3+}$for $\Delta =h_d/\sqrt{3}=34$ MHz. For even and odd multiples of $\pi /4$, a single and a splitted Rabi mode is observed, respectively. The Rabi splitting is the Floquet mode and is constant as a function of $\phi$ but its intensity oscillates with a period of $\pi /2$. The effect is evident in simulations as well, since the terms in $h_{d,i}$ of ${\mathcal {H}}_{RF}$ are along the same direction or orthogonal, for $\phi =0^\circ$ and 45$^\circ$ respectively.

In regards to decoherence sources, it is safe to assume that the main contribution comes from the spin bath made by nuclear spins surrounding the central spin, as well as other electronic spins located in its closed vicinity. Such scenario is the typical situation in spin systems operated at low enough temperatures to reduce the role of the phonon bath on $T_2$. The details of the entangled qubit-bath dynamics are outside the scope of the current study. We do observe that the final results are well described when only dissipation is the source of decoherence, leading to $T_2=2T_1$. This may indicate that the image pulse $h_i$ is able to control the dynamics and thus the decoherence of the spin bath.

The qubit rotation is thus tunable by using a pre-selected value of $\phi$, allowing to create complex rotations. With a decoherence time approaching spin lifetime $T_1$, the value of $\phi$ can be changed while qubit control is still ongoing. Our study demonstrates a sustained quantum coherence using a general protocol that can be readily implemented to any type of qubit. Our approach can be used in other detection schemes, such as sensitive spin detection using on-chip resonance techniques^24–26^.

While preparing our manuscript for resubmission following initial refereeing, a  related protocol applied to NV centers has been published^27^.

## Methods

### Spectrometer setup

The measurements have been performed on a conventional pulse ESR spectrometer Bruker E680 equipped with an incoherent electron double resonance (ELDOR) bridge and a coherent arbitrary waveform generator (AWG) bridge. In the ELDOR bridge (Fig. 1a), the drive and the read out pulses come from two independent sources while with the AWG bridge (Fig.1b) all the pulses are generated using the same microwave source and thus they are all phase coherent. The drive frequency is generated by mixing the source $f_0$ (used as a local oscillator) with a low frequency and phase controllable signal $IF(\Delta ,\phi )$ through an in-phase quadrature (IQ) mixer. Ideally, the output of the mixer is monochromatic with the frequency $f_0+\Delta$. In reality, the output consists of a principal frequency $f_0+\Delta$ (the drive) and of lower amplitude images $f_0+n\Delta$ (see Supplementary Information 1 for more information). Since the effect of the image is the central part of this paper, we have characterized the AWG bridge using a spectrum analyzer, right before the power amplification stage. An example of spectrum is presented in Fig. S6 of the Supplementary Information. The power of the image $f_0-\Delta$ is lower by $\approx -18$ dB than $f_0+\Delta$. Consequently, an amplitude ratio of the MW magnetic fields $h_i/h_d$ around $\sim$0.12 is used in simulations.

### Pulse sequence

First, the system is set to be in resonance condition $g\mu _B H_0=hf_0$. The drive pulse of amplitude $h_d$, frequency $f_0+\Delta$ and length $\tau _{Rabi}$ induces Rabi oscillation in detuning regime. At the same moment the image pulse (generated through the IQ mixer) of amplitude $h_i$, frequency $f_0-\Delta$ and the same length $\tau _{Rabi}$ also irradiate the spins. In order to probe $\left\langle S_z(\tau _{Rabi})\right\rangle$ at the end of the Rabi sequence, we wait a time longer than $T_2$, such that $\left\langle S_x\right\rangle \approx \left\langle S_y\right\rangle \approx 0$, followed by $\pi /2- \pi$ pulses to create a Hahn echo of intensity proportional to $\left\langle S_z\right\rangle$.

### Spin systems

The methodology presented here is demonstrated on different spin systems: the nitrogen substitution in diamond P1 defect ($S=1/2$) (concentration :100 ppm), Mn$^{2+}$ impurities in MgO ($S=5/2$)^28–31^ with a concentration of 10 ppm and Gd$^{3+}$ impurities in CaWO$_4$ ($S=7/2$)^24,32^ with a concentration of 50 ppm. Despite the large Hilbert space of the Mn$^{2+}$ and Gd$^{3+}$ spin Hamiltonians, the orientation of the magnetic field and the frequency and power of the microwave excitation are chosen to avoid multiple level transitions and thus select only one resonance^28^. Therefore, the spin systems can be considered as effective two-level systems undergoing coherent Rabi rotations. The spin Hamiltonians, operating parameters (fields and frequencies) as well as characteristic $T_{1,2}$ times for these materials are given in the Supplementary Information 1.

## Supplementary information

Supplementary Information.

## Data Availability

Data sets generated and analyzed during the current study are available from the corresponding author on request.
