# Author Correction: Experimental protection of quantum coherence by using a phase-tunable image drive

**DOI:** 10.1038/s41598-022-08990-8

**Published:** 2022-03-18

**Authors:** S. Bertaina, H. Vezin, H. De Raedt, I. Chiorescu

**Affiliations:** 1 CNRS, IM2NP (UMR 7334), Institut Matériaux Microélectronique et Nanosciences de Provence, Aix-Marseille Université, 13397 Marseille, France; 2 CNRS, LASIRE (UMR 8516), Laboratoire de Spectroscopie pour les Interactions, la Réactivité et l’Environnement, Université de Lille, 59000 Lille, France; 3 Zernike Institute for Advanced Materials, University of Groningen, Nijenborgh 4, 9747 AG Groningen, The Netherlands; 4 Department of Physics, The National High Magnetic Field Laboratory, Florida State University, Tallahassee, FL 32310 USA

Correction to: *Scientific Reports* 10.1038/s41598-020-77047-5, published online 10 December 2020

The original version of this Article contained an error.

In the Results and discussion section, under the subheading ‘Qubit dynamics’,

“The general condition is $F_{R} = n\Delta ,n \in N$ showing a comensurate motion of the qubit and $h_{i}$ on the Bloch sphere.”

now reads:

“The general condition is $F_{R} = n\Delta ,n = 2k, k \in N$ showing a comensurate motion of the qubit and $h_{i}$ on the Bloch sphere.”

Furthermore, in the Supplementary Information file, in the Coherent pulses in rotating frame: Linear Rabi drive and circularly polarized Qubit protection section, under the subheading ‘Shirley–Floquet formalism’, Equations S18–S21 and surrounding text contained errors. The original Supplementary Information file is provided below.

The original Article and accompanying Supplementary Information file have been corrected.

## Supplementary Information

Supplementary Information.

