# Supplementary material for: Experimental protection of quantum coherence by using a phase-tunable image drive
Source: Sci Rep. 2020 Dec 10;10:21643. doi: 10.1038/s41598-020-77047-5 (PMC7730451; doi:10.1038/s41598-020-77047-5)
Supplement: Supplementary file 1 — Supplementary Information. [file 41598_2020_77047_MOESM1_ESM.pdf]

Experimental protection of quantum coherence by using a phase-tunable image drive. *Supplementary Information*

S. Bertaina,[1,*] H. Vezin,[2] H De Raedt,[3] and I. Chiorescu[4,†]

[1] *CNRS, Aix-Marseille Université, IM2NP (UMR 7334), Institut Matériaux Microélectronique et Nanosciences de Provence, Marseille, France.*

[2] *CNRS, Université de Lille, LASIR (UMR 8516), Laboratoire de Spectrochimie Infrarouge et Raman, Villeneuve d'Ascq, France*

[3] *Zernike Institute for Advanced Materials, University of Groningen, Nijenborgh 4, NL-9747 AG Groningen, The Netherlands*

[4] *Department of Physics, The National High Magnetic Field Laboratory, Florida State University, Tallahassee, Florida 32310, USA.*

(Dated: March 9, 2022)

I. CARR-PURCELL-MEIBOOM-GILL (CPMG) MEASUREMENTS

In the absence of inhomogeneous broadening, the dephasing time or transverse relaxation time T_2^* is directly determined by the ESR linewidth. In solids, and in particular for single crystals, the anisotropic interactions as well as their distribution throughout the crystal induce an inhomogeneity of the line. To measure the dephasing time, the most simple pulse sequence is the Hahn primary echo: a $\pi/2$ pulse rotates the spins in a plane transverse to the static field; due to field inhomogeneity, spin will defocus and spread within the transverse field. A subsequent π pulse reverses spin motion and creates the observed echo signal when refocusing is achieved. By characterizing the exponential decay of the echo signal as a function of the time between the first and the second pulse, one obtains the dephasing time T_2.

However, because of diffusion mechanisms, imperfection of pulses or microwave field inhomogeneity, the refocusing is not complete and the intrinsic dephasing time measured by this sequence is under-evaluated. To reduce these unwanted effects, we used the dynamical decoupling offered by the Carr-Purcell-Meiboom-Gill (CPMG) sequence: after a $(\frac{\pi}{2})_x$ pulse applied along x, a train of π pulses is applied along y with alternate orientations $(\pi_y, \pi_{-y}, \ldots)$. Many primary echoes are thus generated, with an intensity decreasing exponentially as a function of time and characterized by the intrinsic dephasing time T_2. The measurements of T_2 using CPMG sequence for the diamond and $CaWO_4$:$Gd^{3+}$are given in Fig. S1, panels (a) and (b) respectively. Measurements are done at the same temperatures as for the data in the main article Fig. 1 and lead to a T_2 of 0.69 μs and 4 μs respectively. A similar result is obtained if the train of π pulses has alternate orientations along x and y axes $((\pi_x, \pi_y, \pi_{-x}, \pi_{-y}, \ldots)$.

In the case of MgO:Mn^{2+}, the inhomogenous absorption linewidth is only 0.05 mT and the corresponding spin-echo measurement gives a decoherence time of 3 μs (Fig. 2). Since the absorption linewidth is very narrow, the spin-echo signal of MgO:Mn^{2+}has a small amplitude and thus subsequent CPMG pulses are not helpful in this case.

* sylvain.bertaina@im2np.fr

† ic@magnet.fsu.edu

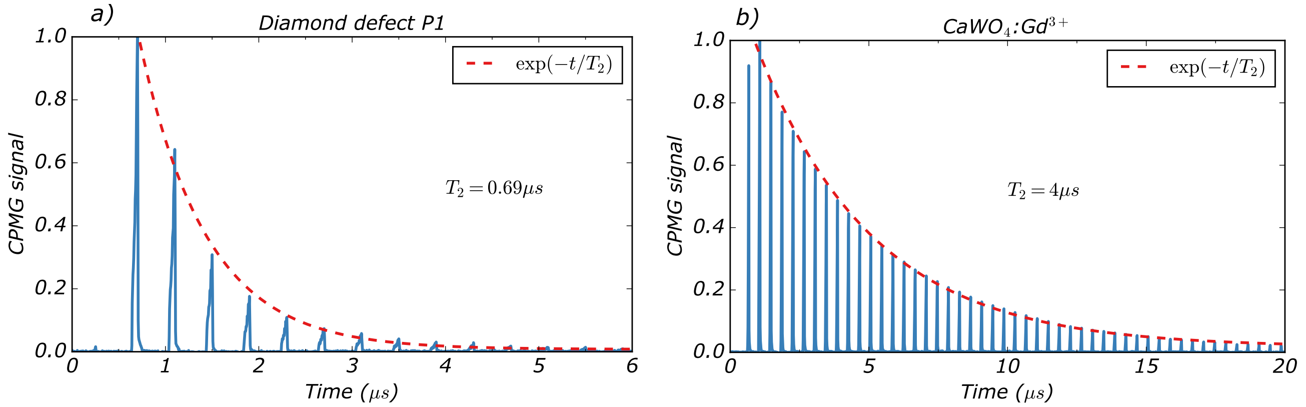

Figure S1. **Coherence times measured with the CPMG protocol.** CPMG signal normalized to the maximum of the echo intensity (blue line) as a function of the total time of the pulse sequence; each peak denotes the addition of a π-pulse into the sequence. The peaks decay following an exponential (red dashed line) with characteristic time T_2. (a) Diamond defect P1 with T_2 = 0.69 μs at T = 15 K. (b) Gd^{3+} spins with T_2 = 4 μs at T = 40 K.

II. SPIN SYSTEMS CHARACTERISTICS

The methodology presented here is applied to different spin systems: the nitrogen substitution in diamond P1 defect ($S = 1/2$) (concentration :100 ppm), Mn^{2+} impurities in MgO ($S = 5/2$) with a concentration of 10 ppm and Gd^{3+} impurities in $CaWO_4$ ($S = 7/2$) with a concentration of 50 ppm. Despite the large Hilbert space of the Mn^{2+} and Gd^{3+} spin Hamiltonians, the orientation of the magnetic field and the frequency and power of the microwave excitation are chosen to avoid multiple level transitions and thus select only one resonance [1]. Therefore, the spin systems can be considered as effective two-level systems undergoing coherent Rabi rotations. The spin Hamiltonians, operating parameters (fields and frequencies) as well as characteristic $T_{1,2}$ times for these materials are given below.

A. P1 defects in diamond

The substituting nitrogen in diamond has covalent bonds to three surrounding carbons and leaves an unpaired electron on the fourth one, giving rise to a spin $S = 1/2$: this is the P1 defect. Its spin Hamiltonian is given by [2]:

$$H_{P1} = g_{P1}\mu_B \vec{B}_0 \vec{S} + \vec{I}[A_N]\vec{S} \tag{S1}$$

where $g_{P1} = 2.0024$ is the g-factor, $I = 1$ is the nuclear spin of ^{14}N and $[A_N]$ is its hyperfine tensor with $A_\perp = 81$ MHz and $A_\parallel = 114$ MHz. A measurement of echo signal as a function of field is shown in Fig. S2. We studied the central line, corresponding to $m_I = 0$, which is not affected by the orientation of the crystal and has the strongest signal. In our experiments, the temperature was set at 15 K, the external field at $B_0 = 343.62$ mT and the microwave frequency at $f_0 = 9.645$ GHz. The concentration of nitrogen is about 100 ppm, which leads to values of the linewidth 2Γ=4 G and dephasing time $T_2 = 0.69\mu s$, similar to values presented in Ref. [3].

B. MgO:Mn^{2+}

MgO:$Mn^{2+}$is the second studied system. The non-magnetic matrix of MgO contains a very low concentration ($\sim$ 10 ppm) of $^{55}Mn^{2+}$ spins with $S = I = 5/2$. The spin Hamiltonian is given by [4]:

$$H_{Mn} = H_{CF} + g_{Mn}\mu_B \vec{B}_0 \vec{S} - A\vec{S}\vec{I} \tag{S2}$$

where $g_{Mn} = 2.0014$ is the g-factor, $A = 244$ MHz is the hyperfine constant and H_{CF} is a crystal field term resulting from the cubic symmetry $F_{m\bar{3}m}$ of MgO: $H_{CF} = a/6[S_x^4 + S_y^4 + S_z^4 - S(S+1)(3S^2+1)/5]$ with $a = 55$ MHz. In previous studies [1, 4–6] we detailed the effect of H_{CF} on the spin eigenvalues, and showed that the static field orientation can tune in-situ their values to be perfect equidistant or non-harmonic. In the present study we operate the static field

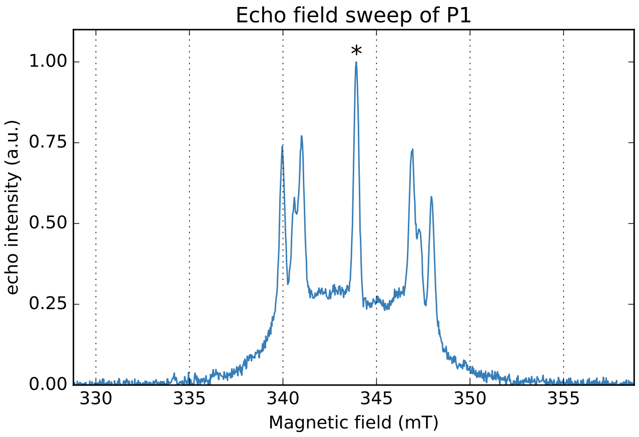

Figure S2. **Echo field sweep of P1**. Echo signal recorded as a function of static field at T=15 K. The most intense line ($m_I = 0$, shown with a star symbol) is not affected by cristal orientation and was selected for the study presented here.

under an alignment generating sufficient non-harmonicity such that one can resonantly select two of the six levels and consider the Mn^{2+} a two-level system. Because of the cubic symmetry and the absence of nuclear spin in the host matrix, the line is very narrow with $2\Gamma = 0.5$ G. In this system the detection of $\langle S_z \rangle$ is not done by echo, but by integrating the area under the Free Induction Decay (FID) signal, measured immediately after τ_{wait} and one $\pi/2$ readout pulse.

The measurements were performed at a temperature of 40 K, with the external field at $B_0 = 353.7$ mT and the microwave frequency $f_0 = 9.734$ GHz.

C. $CaWO_4$:Gd^{3+}

$CaWO_4$:$Gd^{3+}$is the third studied system. Gd^{3+} spins $S = 7/2$ are diluted in non-magnetic matrix of $CaWO_4$ and described by the spin Hamiltonian [7, 8] :

$$H_{Gd} = g_{Gd}\mu_B \vec{B}_0 \vec{S} + B_2^0 O_2^0 + B_4^0 O_4^0 + B_4^4 O_4^4 + B_6^0 O_6^0 + B_6^4 O_6^4 \tag{S3}$$

where $g_{Gd} = 1.991$ and $B_2^0 = -916$, $B_4^0 = -1.14$, $B_4^4 = -7.02$, $B_6^0 = -5.94 \times 10^{-4}$ $B_6^4 = 4.77$ are in MHz units. In our experiments, the temperature was set at 40 K, the external field at $B_0 = 377.0$ mT along to the crystallographic a-axis (tetragonal symmetry $I4/a$) and the microwave frequency at $f_0 = 9.633$ GHz . The full linewidth is 2Γ =6 G A precise analysis of the resonance fields shows a misalignment of about 2.7°. The crystal field anisotropy ensures that the Zeeman levels are not equally spaced, similarly to the case of MgO:Mn^{2+}discussed above. The eigenvalues of H_{Gd} are presented in Fig. S3 (top). The bottom panel shows a typical intensity absorption spectrum at constant frequency as a function of B_0. Peaks appear at resonance fields, indicated by the red segments in the Zeeman diagram (top) which also show the two levels selected for Rabi oscillations. Thus, it is evident that only two levels are involved in the spin dynamics, making the system an effective TLS. Similar type of spectra are measured for the other two samples (diamond and Mn) in order to select the value of the resonance field. The drive and imaging methodology presented here are independent on which resonance is selected .

III. COHERENT PULSES IN ROTATING FRAME: LINEAR RABI DRIVE AND CIRCULARLY POLARIZED QUBIT PROTECTION

A. Generation of coherent pulses using a Hartley mixer

Coherent image and Rabi drives can be obtained with a Hartley mixer having slightly unbalanced RF ports, similar to the one used in our setup. An example of such mixer is shown in Fig. S4 and we will discuss how a circularly polarized image pulse can be constructed. The input radio-frequency (RF) signal is $LO = 2\sin(\omega_{LO}t)$ and therefore each branch will see $LO_1 = \sin(\omega_{LO}t)$ and $LO_2 = \cos(\omega_{LO}t)$. Similarly, $IF = 2A\sin(\omega_{IF}t + \phi_{IF})$. We introduce amplitude and phase mismatches for the IF ports in the following manner: $IF_1 = A_1\cos(\omega_{IF}t+\phi_{IF1})$ and $IF_2 = A_2\sin(\omega_{IF}t+\phi_{IF2})$

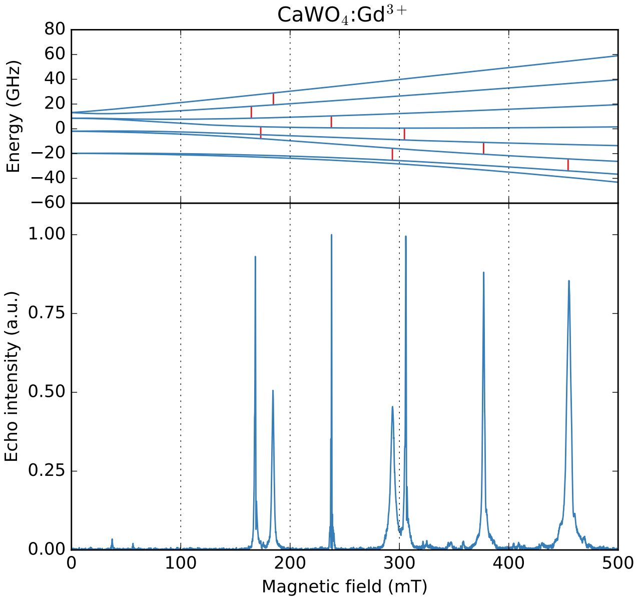

Figure S3. **Absorption spectra for $CaWO_4$:Gd^{3+}.** (top) Energy levels of the Gd^{3+} spin as a function of static magnetic field B_0. The red segments indicate level splittings equal to f_0. (bottom) Absorption intensity spectra (in arbitrary units) as a function of magnetic field obtained by echo field sweep. Peaks appears at the location of the red segments in the top panel. Their intensity depends on the probability to have a transition between the two levels connected by the red segment.

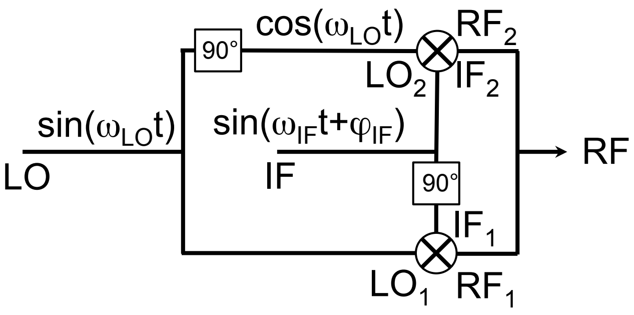

Figure S4. **Hartley mixer.** A schema of a mixer allowing the creation of the Rabi drive h_d and its coherent image h_i, as explained in the text. The main RF drive is at the LO port and it is mixed with a low frequency signal sent into the IF port. The resulting signal exits through the RF port.

and introduce the notations $A = (A_1 + A_2)/2$, $\delta A = (A_1 - A_2)/2$, $\phi = (\phi_{IF1} + \phi_{IF2})/2$, $\delta\phi = (\phi_{IF1} - \phi_{IF2})/2$ and $\omega_\pm = \omega_{LO} \pm \omega_{IF}$. The outputs on the two RF ports are:

$$RF_{1,2} = LO_{1,2} \times IF_{1,2} = \frac{A_{1,2}}{2} \{\sin(\omega_+ t + \phi_{IF1,2}) \pm \sin(\omega_- t - \phi_{IF1,2})\} \tag{S4}$$

and thus

$$\begin{aligned} RF = RF_1 + RF_2 = \frac{A}{2} \{&\sin(\omega_+ t + \phi_{IF1}) + \sin(\omega_- t - \phi_{IF1}) + \sin(\omega_+ t + \phi_{IF2}) - \sin(\omega_- t - \phi_{IF2})\} + \\ \frac{\delta A}{2} \{&\sin(\omega_+ t + \phi_{IF1}) + \sin(\omega_- t - \phi_{IF1}) - \sin(\omega_+ t + \phi_{IF2}) + \sin(\omega_- t - \phi_{IF2})\}. \end{aligned} \tag{S5}$$

We get

$$RF = A\cos\delta\phi\sin(\omega_+ t + \phi) + \delta A\sin\delta\phi\cos(\omega_+ t + \phi) - A\sin\delta\phi\cos(\omega_- t - \phi) + \delta A\cos\delta\phi\sin(\omega_- t - \phi). \tag{S6}$$

with the second term being neglible in first order. The first term represents the Rabi drive pulse with an amplitude $A_d = A\cos\delta\phi$ while the last two show the image pulse which can be rewritten as:

$$\tilde{A}_i(t) = \frac{\delta A\cos\delta\phi}{\cos\theta}\{-\sin\theta\cos(\omega_- t - \phi) + \cos\theta\sin(\omega_- t - \phi)\} = A_i\sin(\omega_- t - \phi - \theta) \tag{S7}$$

with $A_i = \frac{\delta A\cos\delta\phi}{\cos\theta}$ and $\tan\theta = \frac{A\sin\delta\phi}{\delta A\cos\delta\phi} = \frac{\tan\delta\phi}{\delta A/A}$.

B. Effect of the unperfect Hartley mixer on the spin dynamics

To study the qubit dynamics, we write its Hamiltonian first in the laboratory frame and then in the rotating wave approximation. A static magnetic field B_z gives a Zeeman splitting in resonance with $\hbar\omega_{LO}$, while the microwave excitation is $\propto RF(t)S_x$ with $RF(t)$ given by Eq. S6. With notations $\Delta = \frac{\omega_{IF}}{2\pi}$, $f_0 = \frac{\omega_{LO}}{2\pi}$ and $\omega_{+,-} = \omega_{LO} \pm \omega_{IF}$, we have (in units of h):

$$\mathcal{H} = f_0 S_z + 2h_d S_x\sin(\omega_+ t + \phi) + 2h_i S_x\sin(\omega_- t - \phi - \theta), \tag{S8}$$

where $h_{d,i}$, in units of frequency, represent the intensity of the microwave B-fields resulting from the $A_{d,i}$ voltages expressed above; $f_0, h_{d,i}$ and Δ are in units of MHz. Consequently, h_d is the Rabi frequency $F_R = h_d$ resulting from the A_d drive pulse alone.

In a frame rotating with ω_+, the Hamiltonian (in units of h) is transformed into $H_{RF} = U\mathcal{H}U^\dagger + \frac{i}{2\pi}\frac{\partial U}{\partial t}U^\dagger$ with $U = e^{i\omega_+ t S_z}$. Thus:

$$\frac{i}{2\pi}\frac{\partial U}{\partial t}U^\dagger = -\frac{\omega_+}{2\pi}S_z \tag{S9}$$

$$US_xU^\dagger = S_x\cos(\omega_+ t) - S_y\sin(\omega_+ t). \tag{S10}$$

This leads to:

$$\begin{aligned} H_{RF} = -\Delta S_z &+ 2h_d S_x\sin(\omega_+ t + \phi)\cos(\omega_+ t) - 2h_d S_y\sin(\omega_+ t + \phi)\sin(\omega_+ t) \\ &+ 2h_i S_x\sin(\omega_- t - \phi - \theta)\cos(\omega_+ t) - 2h_i S_y\sin(\omega_- t - \phi - \theta)\sin(\omega_+ t) \end{aligned} \tag{S11}$$

and, after rejecting the high frequency terms in $2\omega_+$ and $\omega_+ + \omega_-$,

$$H_{RF} = -\Delta S_z + h_d(S_x\sin\phi - S_y\cos\phi) - h_i[S_x\sin(4\pi\Delta t + \phi + \theta) + S_y\cos(4\pi\Delta t + \phi + \theta)] \tag{S12}$$

or as the real part $H_{RF} = \Re(\mathcal{H}_{RF}) = (\mathcal{H}_{RF} + \mathcal{H}^*_{RF})/2$ of Hamiltonian:

$$\mathcal{H}_{RF} = -\Delta S_z + h_d S_+ e^{-i(\phi - \pi/2)} + h_i S_- e^{-i(4\pi\Delta t + \phi + \pi/2 + \theta)} \tag{S13}$$

or

$$\mathcal{H}_{RF} = -\Delta S_z + S_+[h_d e^{-i(\phi-\pi/2)} + h_i e^{i(4\pi\Delta t + \phi + \pi/2 + \theta)}]. \tag{S14}$$

C. Shirley-Floquet formalism

One can use the Shirley-Floquet formalism as presented in Eq. (7) of Ref.[9] to further analyze the eigenvalues of H_{RF}. The Floquet modes and eigenenergies can be obtained by diagonalizing the operator $\mathcal{K} = H_{RF} - i\partial_t$ with the matrix form:

$$\begin{bmatrix} H_0 & H_1 & H_2 & \dots & \dots & \dots \\ H_{-1} & H_0 - f & H_1 & \dots & \dots & \dots \\ H_{-2} & H_{-1} & H_0 - 2f & \dots & \dots & \dots \\ \vdots & \vdots & \vdots & \ddots & \vdots & \vdots \\ \vdots & \vdots & \vdots & \dots & H_0 - nf & \dots \\ \vdots & \vdots & \vdots & \dots & \vdots & \ddots \end{bmatrix} \tag{S15}$$

where $H_n = \frac{1}{\tau}\int_0^\tau H_{RF}(t)e^{i2\pi nft}dt$, $\tau = f^{-1}$ and $f = 2\Delta$.

H_{RF}'s first two terms are time independent and thus form the H_0 component, while the third one gives the terms $n = \pm 1$:

$$H_0 = -\Delta S_z + \frac{h_d}{2}[S_+ e^{-i(\phi-\pi/2)} + S_- e^{i(\phi-\pi/2)}], \tag{S16}$$

$$H_{\pm 1} = \frac{h_i}{2} S_\mp e^{\mp i(\phi+\pi/2+\theta)}. \tag{S17}$$

The spin systems studied here behave as two-level systems and therefore one assumes $S = 1/2$ in the matrix above:

$$H_{SF} = \frac{1}{2}\begin{bmatrix} \Delta & h_d e^{-i(\phi-\pi/2)} & 0 & 0 & 0 & 0 & \dots \\ h_d e^{i(\phi-\pi/2)} & -\Delta & h_i e^{-i(\phi+\pi/2+\theta)} & 0 & 0 & 0 & \dots \\ 0 & h_i e^{i(\phi+\pi/2+\theta)} & \Delta - 4\Delta & h_d e^{-i(\phi-\pi/2)} & 0 & 0 & \dots \\ 0 & 0 & h_d e^{i(\phi-\pi/2)} & -\Delta - 4\Delta & h_i e^{-i(\phi+\pi/2+\theta)} & 0 & \dots \\ 0 & 0 & 0 & h_i e^{i(\phi+\pi/2+\theta)} & \Delta - 8\Delta & h_d e^{-i(\phi-\pi/2)} & \dots \\ 0 & 0 & 0 & 0 & h_d e^{i(\phi-\pi/2)} & -\Delta - 8\Delta & \dots \\ \vdots & \vdots & \vdots & \vdots & \vdots & \vdots & \ddots \end{bmatrix} \tag{S18}$$

with Δ and h_d of comparable magnitudes and much larger than h_i. In the limit $h_i \to 0$, we can replace the 2×2 diagonal blocks with a diagonal $[F_R/2, -F_R/2, F_R/2 - 2\Delta, -F_R/2 - 2\Delta, F_R/2 - 4\Delta, -F_R/2 - 4\Delta, \dots]$ and zero-ed out subdiagonals, with:

$$F_R = \sqrt{\Delta^2 + h_d^2}. \tag{S19}$$

The condition for the image pulse h_i to sustain the coherence between even- and odd-numbered quasi-energies is therefore $-F_R/2 = F_R/2 - n\Delta$ or

$$F_R = n\Delta \text{ and } \sqrt{\Delta^2 + h_d^2} = n\Delta \text{ with } n = 2k, k \in N \tag{S20}$$

In our study, $n = 2$ and the coupling is done between the second and third element of the diagonal, via off-diagonal terms containing both h_d and h_i. The splitted eigenvalues of the Shirley-Floquet Hamiltonian are ϕ-independent. Experimentally, the off-diagonal coupling is larger than the linewidth of the Rabi modes, such that a splitting is observed.

An analytical estimation of H_{SF} eigenvalues can be done using the second order perturbation theory for the 6x6 block shown in Eq. S18. The determinant $\det[H_{SF} - \lambda I] = 0$ with I the identity matrix and λ representing the eigenvalues, is expanded with Mathematica in powers of h_i and equated to zero up to h_i^4. For the levels involved in the $n = 2$ resonance, the eigenvalues are given by:

$$E_\pm = -\Delta \mp \frac{3}{8}h_i + \frac{7h_i^2}{512\Delta}. \tag{S21}$$

The splitting can thus be estimated as $E_+ - E_- = -\frac{3h_i}{4}$.

D. Torque considerations

The qubit dynamics imposed by Hamiltonian H_{RF} can be simulated using `QuTIP` [10] as discussed in the main article. Here, we supplement the understanding of qubit dynamics from the point of view of spin's $\vec{S}$ torque in two situations, $\phi = 0$ and $\pi/4$ (see Fig. S5 *a* and *b*, respectively) in absence of decoherence. Starting from ground state and for a Rabi frequency of 20 MHz, we compute torque magnitudes for the drive and image pulses as a function of time, $|\vec{S} \times \vec{h_\delta}|(t)$ and $|\vec{S} \times \vec{h_i}|(t)$ respectively, with $\vec{h_\delta} = \vec{h}_d + \Delta\hat{z}$. For simplicity and without loss of generality, the phase θ, an angular shift of the initial phase of h_i, is assumed equal to zero. For comparison, the resonance case at $\Delta = h_i = 0$ is shown as an horizontal dashed line with maximum torque value since the spin and the microwave field are orthogonal during the Rabi nutation. All other values are normalized to this maximum value. The zero

torque dashed line represents the case of "spin locking" when $\vec{S}||h_\delta$. The zero-torque concept of spin-locking applies independently on detuning Δ but it is valid only if $h_i \equiv 0$; otherwise, the image pulse pulls the spin off the axis of $\vec{h}_\delta$.

In contrast to the spin locking case, both torques are non-zero for our protection protocol. The drive torque at resonance $h_d = \Delta\sqrt{3}$ (in red) is performing the Rabi nutation needed for gate operations while the image torque (in blue) acts as a small perturbation; in this example h_i is 5% of h_d or -26dB in power. The effect of the initial phase ϕ of h_i is essential to qubit dynamics although h_i remains a small perturbation during the gate operation. When the initial torques are parallel or antiparallel ($\phi = 0$ or $\pi/2$ respectively) the Rabi rotation is closer to full amplitude and the Floquet mode is less visible. This is shown by a small modulation in drive torque (in red, panel *(a)*) with the period of the Floquet mode. In contrast, when torques are initially perpendicular to each other ($\phi = \pi/4$, panel *(b)*), the Rabi nutation and drive torque has strong beatings, while retaining the same amount of coherence protection. The Floquet mode is clearly visible in this case, as discussed in Fig. 4 of the main article.

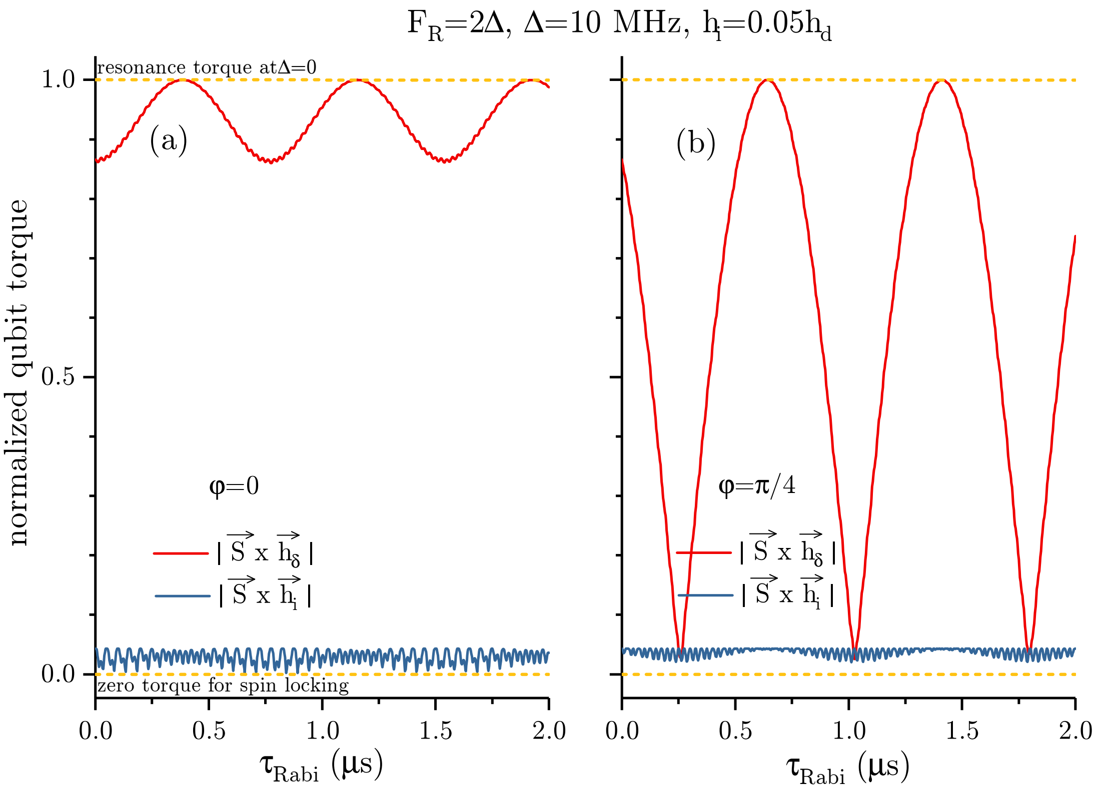

Figure S5. **Qubit torque during Rabi oscillations.** Torque values are normalized to the maximum value for $\Delta = 0$ (top orange dash line) and calculated for the total drive $\vec{h}_\delta$ and the image $\vec{h}_i$ pulses (red and blue lines respectively), starting from the initial ground state. The case of spin-locking is shown by the zero torque dashed line. The qubit dynamics is highly sensitive to the initial phase of the image pulse: (a) $\phi = 0$, initial torques are parallel and Rabi oscillations show one main frequency; (b) $\phi = \pi/4$, initial torques are orthogonal and a Floquet mode is generated as a strong beating of the Rabi frequency (see also Fig. 4 of the main article).

IV. CALIBRATION OF THE MICROWAVE FIELDS

The method presented in this article is highly dependent on the value of the microwave fields h_d and h_i. The drive intensity h_d is easy to calibrate by measuring the Rabi frequency at resonance while knowing its expected value from the spin Hamiltonian in the rotating frame ($\Delta = 0$).

On the contrary, h_i is more difficult to calibrate. As seen in Section III A, h_i comes from the inherent unbalance

of a real mixer and therefore it can be device depended. To calibrate h_i, we have used a spectrum analyzer Agilent Technologie, PXA Signal Analyzer N9030A. We have measured the Fourier transform of the microwave coming from the AWG bridge before the power amplification stage as shown in Fig. S6: f_0 is the carrier frequency, $f_0 + \Delta$ is the Rabi drive frequency (h_d) and $f_0 - \Delta$ is the image frequency. The image pulse is about 100 times ($\sim$ 18 dB) weaker than the Rabi drive and thus it can be used to sustain the motion rather than driving it. The signal at f_0, about 4-6 dB smaller than the image drive, is not in a Floquet resonance with the Rabi drive (see Eq. S20) and thus not contributing to spin dynamics.

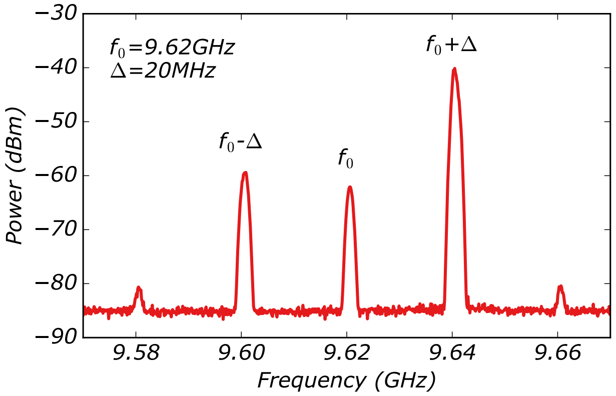

Figure S6. **Power spectra of the RF signal.** Spectrum analyzer data showing the RF power spectra, after the mixer shown in Fig. 1 of the main article. The Rabi drive pulse is located at $f_0 + \Delta$, $\sim$ 18 dB stronger than the image pulse used for qubit protection, located at $f_0 - \Delta$.

[1] S. Bertaina, L. Chen, N. Groll, J. Van Tol, N. S. Dalal, and I. Chiorescu, Phys. Rev. Lett. **102**, 50501 (2009).
[2] W. V. Smith, P. P. Sorokin, I. L. Gelles, and G. J. Lasher, Phys. Rev. **115**, 1546 (1959).
[3] J. A. van Wyk, E. C. Reynhardt, G. L. High, and I. Kiflawi, J. Phys. D: Appl. Phys. **30**, 1790 (1997).
[4] S. Bertaina, G. Yue, C.-E. Dutoit, and I. Chiorescu, Phys. Rev. B **96**, 024428 (2017).
[5] S. Bertaina, N. Groll, L. Chen, and I. Chiorescu, J. Phys. Conf. Ser. **324**, 012008 (2011).
[6] S. Bertaina, M. Martens, M. Egels, D. Barakel, and I. Chiorescu, Phys. Rev. B **92**, 024408 (2015).
[7] C. Hempstead and K. Bowers, Phys. Rev. **118**, 131 (1960).
[8] G. Yue, L. Chen, J. Barreda, V. Bevara, L. Hu, L. Wu, Z. Wang, P. Andrei, S. Bertaina, and I. Chiorescu, Appl. Phys. Lett. **111**, 202601 (2017).
[9] A. Russomanno and G. E. Santoro, J. Stat. Mech. Theory Exp. **2017**, 103104 (2017).
[10] J. Johansson, P. Nation, and F. Nori, Comput. Phys. Commun. **184**, 1234 (2013).
